# Variant-dependent oxidative and cytokine responses of human neutrophils to SARS-CoV-2 spike protein and anti-spike IgG1 antibodies

**DOI:** 10.3389/fimmu.2023.1255003

**Published:** 2023-10-16

**Authors:** Nathalie Bonatti Franco Almeida, Kayla Marie Fantone, Demba Sarr, Nuha Milad Ashtiwi, Sarah Channell, Rafaella Fortini Queiroz Grenfell, Olindo Assis Martins-Filho, Balázs Rada

**Affiliations:** ^1^ Department of Infectious Diseases, The University of Georgia, Athens, GA, United States; ^2^ René Rachou Institute, Oswaldo Cruz Foundation, Belo Horizonte, Minas Gerais, Brazil

**Keywords:** SARS-CoV-2, spike protein, neutrophil, anti-spike IgG1, immune complex, influenza, ROS, MPO

## Abstract

**Introduction:**

Severe forms of COVID-19, the disease caused by SARS-CoV-2, are characterized by acute respiratory distress syndrome, robust lung inflammation and death in some patients. Strong evidence has been accumulating that polymorphonuclear neutrophilic granulocytes (PMN) play an important role in the pathophysiology of severe COVID-19. SARS-CoV-2 directly induces *in vitro* PMN activation, mainly the release of neutrophil extracellular traps (NETs). However, the viral components inducing this PMN response remain unclear.

**Methods:**

In this work human PMN responses were assessed *in vitro* in response to the spike (S) protein of two different SARS-CoV-2 variants, anti-S IgG1 antibodies or immune complexes formed by them. Production of reactive oxygen species (ROS) was measured by Diogenes-based chemiluminescence. Release of myeloperoxidase (MPO) was assessed by ELISA while secretion of a list of cytokines and growth factors was determined by high-performance multiplex cytokine assay.

**Results and discussion:**

We show that the SARS-CoV-2 Omicron variant S protein and anti-spike IgG1, either alone or together, stimulate ROS production in human PMNs. We also observed that the SARS-CoV-2 Wuhan S protein and anti-S IgG1 antibody together trigger MPO release from PMNs. Based on the relevance of SARS-CoV-2 and influenza co-infections, we have also investigated the impact of influenza virus infection on the previous PMN responses to S proteins or anti-S antibodies. We did not detect any significant effect of influenza co-infection on ROS generation in PMNs. Our data also show that PMN stimulation by S proteins induced the release of different chemokines, growth factors, regulatory and proinflammatory cytokines. Overall, our findings show that the SARS-CoV-2 S protein, an anti-spike IgG1 antibody or their immune complex, promote oxidative responses of PMNs in a variant-dependent manner, contributing to a better understanding of the role of PMN responses during SARS-CoV-2 infection.

## Introduction

A novel and highly pathogenic coronavirus emerged in late 2019 in Wuhan, China (1). The virus was named severe acute respiratory syndrome coronavirus-2 (SARS-CoV-2) (2). SARS-CoV-2 caused a large number of respiratory diseases around the world, termed Coronavirus Disease 2019 (COVID-19), and was defined as a pandemic in March 2020 by the World Health Organization (WHO) (3). SARS-CoV-2 has structural proteins that include the spike (S) protein, the envelope (E) protein, the membrane (M) protein, and the nucleocapsid (N) protein (4). The S protein is a major surface antigen of SARS-CoV-2 and represents an important target for vaccines and therapeutics (4). The S protein has a host receptor-binding domain (RBD) (S1) and membrane-fusion (S2) subunits (4). Furthermore, the SARS-CoV-2 S protein binds the angiotensin-converting enzyme 2 (ACE-2) receptor for cellular entry (5). Over time, several new SARS-CoV-2 variants of concern emerged and were reported to the WHO (6). The Omicron variant is the most transmissible variant of the SARS-CoV-2 and is considered a variant of concern by WHO (6). The omicron S protein has mutations in the RBD region, and this contributes to immune evasion of SARS-CoV-2 (7). SARS-CoV-2 infection can cause acute respiratory distress syndrome (ARDS) that represents respiratory failure and alveolar damage in the lungs and has been associated with severe COVID-19 (8). ARDS originates from dysregulated, local, and systemic immune activation and leads to pulmonary vascular, parenchymal, and alveolar damage (8). Activation of polymorphonuclear neutrophil granulocytes (PMNs), innate immune cells crucial for cell-mediated defense against microbes, have been linked to ARDS in COVID-19 (8). Oxidative stress markers derived from PMNs were reported to be elevated in patients with COVID-19 (9). The production of reactive oxygen species (ROS) is a key signaling phenomenon to the progression of many inflammatory diseases (9). In several infectious diseases, including respiratory viral infections, ROS are produced by cells, such as PMNs (9). Upon PMN activation, myeloperoxidase (MPO) is released extracellularly in the circulation or tissues and have been shown to be associated with severe COVID-19 (10).

Immunoglobulins (Ig) are important effector components of the adaptive immune response (11). The IgG subclasses are highly effective in neutralizing viral particles and mediate viral clearance. However, it has been shown that IgG immune complexes (ICs) with viral particles can aggravate COVID-19 (11). IgG is known to bind to constant fragment Fc gamma receptors (FcγRs) and exerts effector functions on immune cells including PMNs (12). The complex of IgG antibody with SARS-CoV-2 S protein was reported to induce an inflammatory response in macrophages from COVID-19 patients (13).

Previous reports have demonstrated that SARS-CoV-2 and influenza A virus (IAV) co-infection occurs worldwide. However, more studies are needed to better comprehend the implications and outcomes of these co-infections (14). While emerging results indicate that SARS-CoV-2 virus exposure leads to PMN activation *in vitro*, it remains unknown which components of the virus are responsible for this (8, 9).

In this study, we have assessed *in vitro* how PMNs respond to the S1 proteins of the Wuhan and the Omicron variants of SARS-CoV-2, anti-S IgG1 antibodies, and the ICs formed by them. We have also investigated whether exposure of PMNs to IAV affects the aforementioned functions of PMNs.

## Methods

### Study participants and sample collection

Healthy blood donors (*n* = 30) were recruited at the Health Center or the Clinical Translational Research Unit at The University of Georgia (UGA). Before blood donation, the volunteers provided and signed informed consent forms according to the UGA IRB-approved protocol #UGA 2012-10769.

### Neutrophil isolation

Human PMNs were isolated using the EasySep™ Direct Human Neutrophil Isolation Kit (catalog #19666, Stem Cell Technologies, Vancouver, BC, CA). Briefly, 30 mL of blood were drawn into EDTA-coated tubes and proceeded with the protocol according to the manufacturer’s instructions. This protocol purified PMNs by negative isolation, ensuring minimal activation of the cells, and yielded 30–130 × 10^6^ live PMNs with >98% viability as assessed by Trypan Blue staining and 95% purity as determined by cytospin preparations or flow cytometry.

### Serum samples

Eight milliliters of venous blood were drawn from healthy human volunteers by venipuncture into a tube without anticoagulant. After clotting, the tubes were centrifuged at 2,000 × g for 5 min and the supernatant was either stored frozen (−80°C) or immediately used for experimentation. The serum was used to prepare assay medium consisting of 1X HBSS, 10 mM HEPES, 5 mM glucose, and 1% (v/v) autologous serum. This assay medium was used in all experiments.

### Recombinant proteins and monoclonal antibody

The Wuhan S1 protein (catalog #MBS9719012) was purchased from MyBioSource (San Diego, CA, USA). The Omicron S1 protein (catalog #S1N-C52Ha) was generously donated by ACRO Biosystems (Newark, DE, USA). The spike coding sequence includes the Omicron variant (B.1.1.529). Both recombinant proteins were tested for endotoxin, and the results showed < 1 EU per μg of the protein as determined by the Limulus amoebocyte lysate method.

Humanized IgG1 anti-S protein antibody (catalog #CR3022) was purchased from Cell Signaling Technology (Danvers, MA, USA). Five different concentrations (0.005, 0.05, 0.5, 5, or 10 μg/mL) of S1 protein or the anti-S antibody were tested and used to stimulate PMNs. The monoclonal antibody was derived from a monoclonal antibody generated semi-synthetically from a patient infected with SARS-CoV-2. The antibody is a human neutralizing antibody (hNAb) and binds SARS-CoV-2 S proteins of lineages B.1.1.7 (alpha), B.1.351 (beta), P.1 (gamma), B.1.617.2 (delta), B.1.429 (epsilon), B.1.525 (eta), and B.1.617.1 (kappa). S1 protein (10 μg/mL) promotes activation profile on human dendritic cells. To observe a potential additive effect of S1 and anti-S IgG1 (possibly via IC formation), Wuhan and Omicron S1 proteins and the anti-S IgG1 were first incubated at 37°C for 30 min before they are used for stimulation of PMNs. S1 protein or the antibody alone was also tested. Protein stock was diluted in sterile phosphate-buffered saline (PBS).

### Influenza virus

Seven different multiplicities of infection (MOIs) of the influenza virus strain A/Hong Kong/8/1968 (H3N2; NR-346) were tested (0.1, 0.3, 0.5, 1, 3, 6, and 10). The virus was purchased from BEI Resources (managed by the American Type Culture Collection [ATCC]), NIH. The virus propagation was performed in Madin–Darby canine kidney (MDCK) cells. The virus was cultured in MDCK cells (ATCC, CCL034) using an infection medium (DMEM/F-12 supplemented with 1 mM of L-glutamine with 1 mg/mL of tosylsulfonyl phenylalanyl chloromethyl ketone-treated trypsin). The virus was harvested 48 h post-infection. Viral titer was determined by plaque-forming unit (PFU) assay (5 × 10^7^ PFU/mL).

### Cell viability

A total of 10^6^ PMNs were stained with a Zombie Aqua Fixable Viability Kit (catalog #423102, BioLegend, San Diego, CA, USA) at a dilution of 1:10,000 for 15 min at room temperature (RT). Cells were washed with PBS and centrifuged at 400 × *g* for 10 min. Cells were suspended in PBS containing 1% bovine serum albumin (PBS-BSA) and incubated with human Fc blocker (catalog #422303, BioLegend, San Diego, CA, USA) for 10 min at RT. Cells were centrifuged as described previously. Cells were labeled using antibodies targeting these surface markers: CD66b conjugated to FITC fluorochrome (catalog #305104, BioLegend, San Diego, CA, USA) and CD11b conjugated to PECy7 fluorochrome (catalog #561685, BD, Franklin Lakes, NJ, USA) for 30 min at RT, protected from light. Cells were washed in PBS- BSA 1%, centrifuged, and resuspended in 300 µL of BD Stabilizing Fixative (catalog #338036, BD, Franklin Lakes, NJ, USA). The samples were analyzed at the UGA College of Veterinary Medicine Cytometry Core Facility using the NovoCyte Quanteon (Agilent, Santa Clara, CA, USA). Samples were analyzed using the NovoSamplerQ utilizing NovoExpress software v.1.4.1 (Agilent, Santa Clara, CA, USA).

### Reactive oxygen species assay

Five different concentrations of the recombinant S1 proteins, the anti-S IgG1, and their ICs were used to stimulate PMNs *in vitro*. In all experiments, unstimulated PMNs and PMNs treated with 100 nM of phorbol-12-myristate-13-acetate (PMA) were used as negative and positive controls, respectively. PMA is a molecule that induces potent activation of PMNs via protein kinase C activation. ROS production of PMNs was measured by Diogenes chemiluminescence (National Diagnostics, Atlanta, GA, USA) using the Varioskan Flash microplate luminometer (Thermo Scientific, Waltham, MA, USA). Briefly, 250,000 PMNs in assay media were stimulated with the molecules previously mentioned. Relative luminescence units (RLUs) were measured for 120 min. Total ROS generation during the indicated duration of the measurement was calculated based on accumulated luminescence and normalized using the PMA-stimulated signal as 100%.

### Enzyme-linked immunosorbent assay for detection of MPO

A total of 1 × 10^6^ PMNs were stimulated with S1 protein, the anti-S IgG1 or their ICs (10 μg/mL), and IAV (1 MOI) at 37°C for 3 h. The incubation was carried out in microtubes in a volume of 100 μL per condition. Following incubation, cells were centrifuged at 400 × *g* for 10 min. The supernatants were diluted 1:200 in PBS-BSA 1% before being added to the plate. Release of human MPO from PMNs was measured using a commercial enzyme-linked immunosorbent assay (ELISA) kit (catalog #DY3174, R&D Systems, Minneapolis, MN, USA) according to the manufacturer’s instructions. Standard curves were used to determine the concentration of MPO in each sample. The results were reported in picograms per milliliter (pg/mL).

### Multiplex cytokine assay

PMN supernatants were diluted according to the manufacturer’s instructions, and 50 μL was used to quantify the levels of 27 analytes, namely, chemokines (CCL11, CXCL8, CCL3, CCL4, CCL2, CCL5, and CCL10), proinflammatory cytokines (IL-1*β*, IL-6, TNF-α, IL-12, IFN-γ, IL-15, and IL-17), regulatory cytokines (IL-1Ra, IL-4, IL-5, IL-9, IL 10, and IL-13) and growth factors (FGF-basic, PDGF, VEGF, G-CSF, GM-CSF, IL-2, and IL7), by high-performance multiplex assay Bio-Plex Pro Human Chemokine 27-plex assay (catalog #M500KCAF0Y, Bio-Rad, Hercules, CA, USA). Biomarkers were assessed simultaneously using the Bio-plex 3D suspension array system (Bio-Rad, Hercules, CA, USA). The concentration of each analyte was calculated based on standard curves and analyzed by Bio- Plex Manager software version 6.1 (Bio-Rad, Hercules, CA, USA). The biomarkers’ concentrations were expressed as pg/mL.

### Statistical analysis

Statistical analysis and graphical data presentations were performed using GraphPad Prism version 8.0.2. Median was plotted on a box plot graph. Statistical differences were determined using the Friedman test and Dunn’s multiple comparisons test to compare groups on ROS assays. Unpaired *t*-test or Mann–Whitney test were used to analyze the Luminex data. Statistical differences were determined using the Mann–Whitney test to compare groups. Statistical significance was denoted as *p < 0.05, **p < 0.01, ***p < 0.001, and ****p < 0.0001, and *p* > 0.05 was considered not significant (ns). The overall signature of soluble mediators in PMN supernatants was assembled by converting the raw data, expressed in pg/mL, into categorical results to determine the proportion of subjects above the global median cutoff calculated for each analyte. Increased proportion of subjects were considered for values above the 50th percentile.

## Results

We first assessed PMN viability and purity after isolation from human blood by flow cytometry. Cells were gated on live PMNs using Zombie Aqua-A dye. PMNs were considered CD66b^+^/CD11b^+^ (double positive) and the protocol yielded PMNs with ~95% purity (mean 95.52 ± 5.27). Representative flow cytometry plots illustrating the PMN viability and purity are provided in **Supplementary Figure 1**.

**Figure 1:**
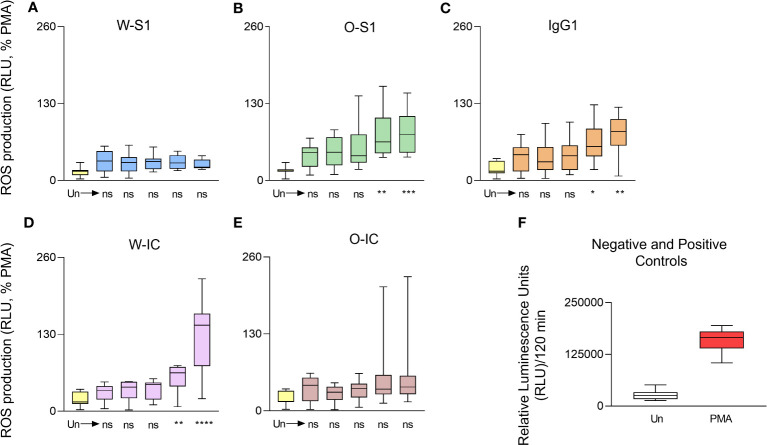
Dose dependence of ROS generation of human PMNs in response to SARS- CoV-2 S1 proteins, anti-S IgG1, and their immune complexes. Human PMNs were stimulated with Wuhan S1 protein **(A)**, Omicron S1 protein **(B)**, the anti-S1 IgG1 **(C)**, the Wuhan immune complex (IC) **(D)**, or the Omicron-IC (**E**, O-IC) at the indicated doses (0.005, 0.05, 0.5, 5.0, and 10.0 μg/mL). ROS production was assessed for 2 h post-stimulation using the Diogenes ROS-detecting chemiluminescence kit. Total ROS production was calculated for the entire duration of the measurement (2 h), normalized on the ROS signal obtained in the case of the positive control (PMA stimulation) and expressed as the percentage of the PMA-stimulated ROS signal. In case of IC stimulation, each component (S1 or IgG1) was added at the same, indicated dose. The Wuhan S1 protein (W-S1) did not significantly affect ROS generation by PMNs (*n* = 9) while Omicron S1 (O-S1) did (5 or 10 μg/mL: **p = 0.0023 and *** *p* = 0.008, respectively, *n* = 8); 5 or 10 μg/mL doses of IgG1 induced significant ROS production by PMNs (*n* = 10, *p = 0.0143 and **p = 0.0029, respectively). The Wuhan immune complex (W-IC) significantly enhanced ROS generation in PMNs at doses of 5 or 10 μg/mL (**p = 0.0071 and **** p < 0.0001, respectively, *n* = 10). The Omicron immune complex (O-IC) did not significantly affect the ROS generation by PMNs (*n* = 9). **(F)** The raw ROS data for the negative (Un: unstimulated) and positive (PMA, phorbol myristate acetate) controls are shown (mean ± S.D., *n* = 8). Ns, nonsignificant; RLU, relative luminescence unit; ROS, reactive oxygen species.

Our next goal was to examine ROS generation by PMNs in response to S protein and its ICs because ROS represents an essential effector function of these cells and are also significant contributors to lung damage in several respiratory infections including COVID-19. We used five different concentrations (0.005, 0.05, 0.5, 5.0, and 10.0 μg/mL) of the S1 proteins, the anti-S IgG1, or their ICs to stimulate PMNs and measured ROS production. PMNs responded with a dose-dependent increase in ROS production when stimulated with increasing doses of either the Omicron S1 protein, anti-S IgG1, or the Wuhan IC. No significant differences could be observed in the ROS response with stimulation of the Wuhan S1 protein (**Figure 1**). PMNs produced significantly more ROS when exposed to 5 or 10 μg/mL of Omicron S1 protein (**Figure 1**). PMNs stimulated with 5 or 10 μg/mL of anti-S IgG1 produced significantly more ROS (**Figure 1**). The Wuhan IC (5 or 10 μg/mL of each component) significantly enhanced ROS generation in PMNs (**Figure 1**). However, no significant differences could be observed in the ROS response between the stimulation with Omicron IC versus unstimulated PMNs (**Figure 1**).

**Figure 2:**
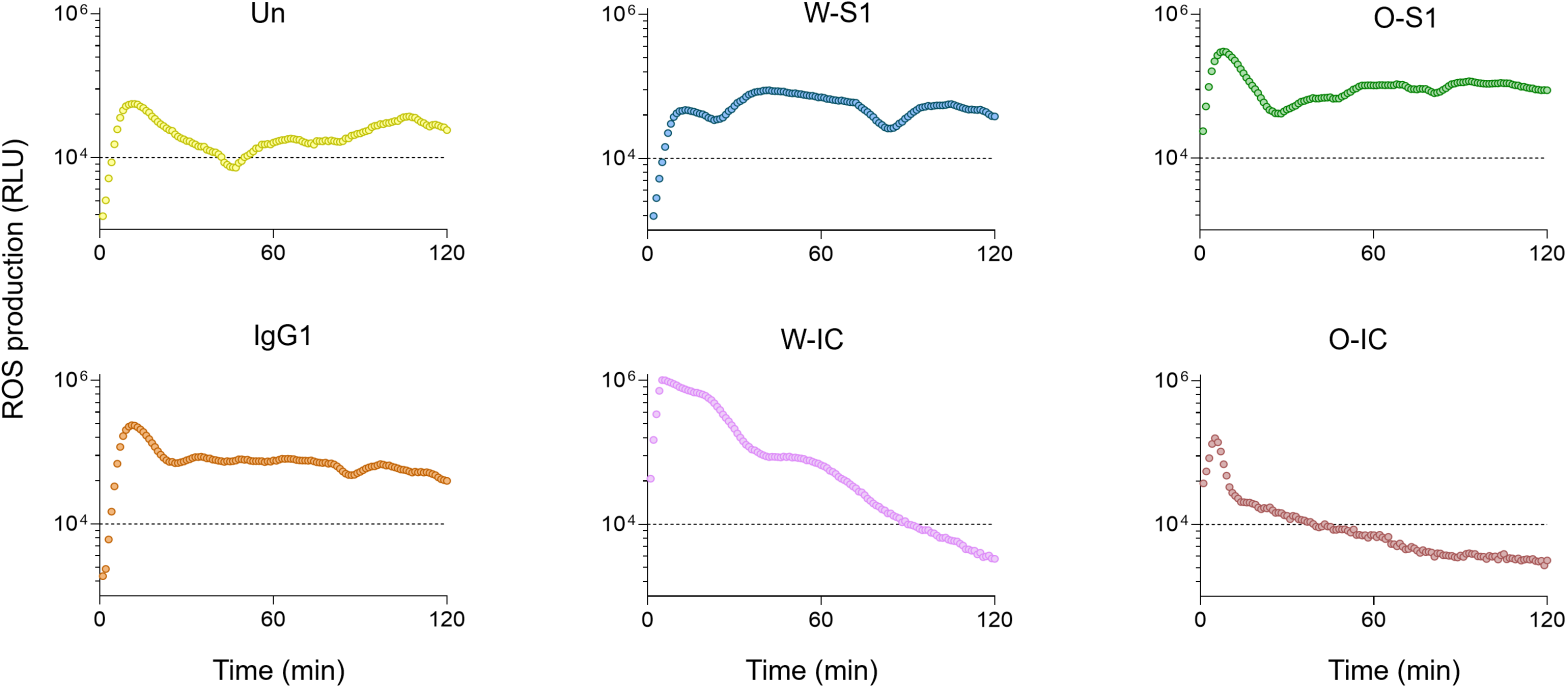
The kinetics of ROS release in human PMNs. Representative ROS kinetics of eight similar results from independent PMN donors are shown. Human PMNs were exposed to different stimulants: Wuhan Spike 1 (W-S1, blue), Omicron Spike 1 (O-S1, green), IgG1 (orange), Wuhan-immune complex (W-IC, pink), and Omicron immune complex (O-IC, brown) and compared to unstimulated PMNs (Un, yellow). ROS production was measured for 2 h using the Diogenes ROS-detecting chemiluminescence kit. PMA, phorbol myristate acetate; RLU, relative luminescence unit; ROS, reactive oxygen species.

**Figure 3:**
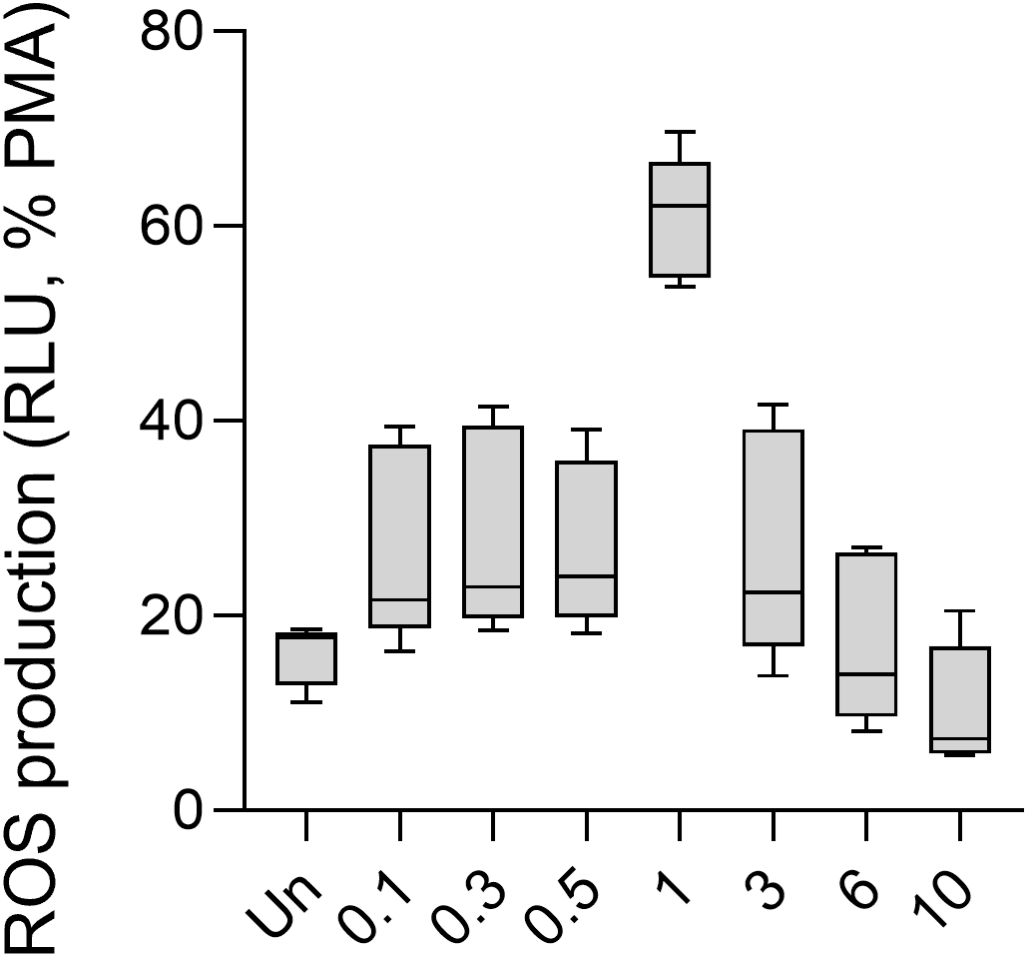
ROS production of human PMNs in response to different doses of the influenza virus. Human PMNs were stimulated with different multiplicity of infections (MOI) of the IAV strain A/Hong Kong/8/1968 (H3N2). Unstimulated and 100 nM PMA-treated PMNs were used as controls. ROS production was assessed using the Diogenes chemiluminescence assay (*n* = 5). PMA, phorbol myristate acetate; RLU, relative luminescence unit; ROS, reactive oxygen species.

When we next compared the results of ROS production of PMNs stimulated with the highest concentrations (10.0 μg/mL) of S1 or anti-S1 IgG1, enhanced ROS release was observed compared to unstimulated PMNs (**Figure 2**). The Omicron S1 resulted in a significantly higher ROS signal in PMNs than Wuhan S1 that resulted in a significant but minor ROS output in PMNs (**Figure 2**). The anti-S1 IgG1 (mAb) alone also stimulated ROS production in human PMNs (**Figure 2**). ROS generation triggered by the Wuhan IC was significantly higher than that stimulated by Wuhan S1 alone (**Figure 3**). Interestingly, ROS production by PMNs in the presence of the Omicron IC was significantly lower than the signal by Omicron S1 or IgG1 alone (**Figure 2**). These results indicate that while human PMNs respond to both S1 variants with ROS release, their response to the ICs is variant-dependent.

As mentioned previously, co-infection between SARS-CoV-2 and IAV H3N2 occurs frequently. The H3N2 IAV virus is the most resistant to anti-influenza drug therapies (14). Therefore, to explore whether the oxidative responses of PMNs to S1, the anti-S1 IgG, or their ICs described above are affected by influenza infection, in our experiments, we infected human PMNs with the A/Hong Kong/8/1968 H3N2 influenza virus and measured ROS production in response to S1, anti-S1 IgG1, and their ICs (15). We first analyzed the ROS response of PMNs to influenza virus alone. We observed an increasing trend in ROS production 0–1 MOI that became approximately 1–10 MOI (**Figure 3**). Only 1 MOI showed significantly higher ROS production by PMNs than unstimulated cells (**Figure 3**). Thus, human PMNs respond to IAV in a dose-dependent manner with ROS production.

**Figure 4:**
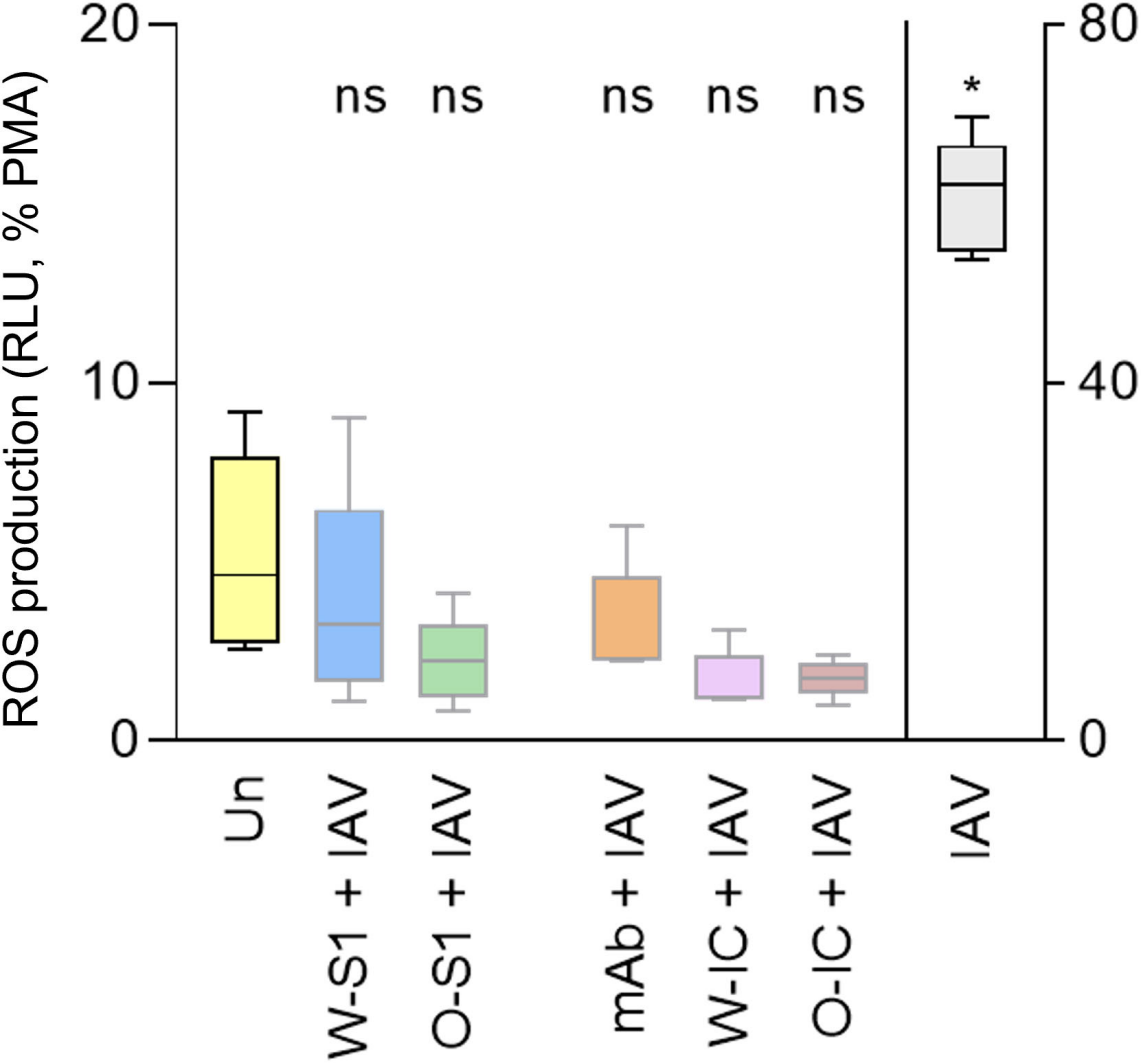
ROS generation by human PMNs in response to Wuhan and Omicron S1 proteins of SARS-CoV-2, anti-S IgG1 monoclonal antibody, and their ICs, in the presence of influenza. Human PMNs were left unstimulated (Un) or stimulated with 1 MOI of the influenza A virus A/Hong Kong/8/1968 (H3N2) strain (IAV). PMNs were also co-stimulated with the Wuhan or Omicron S1 protein (W-S1, O-S1, 10 μg/mL of protein), the anti-spike IgG1 (mAb, 10 μg/mL of antibody), or their immune complexes (W-IC or O-IC) as indicated. PMN (100 nM) was used as a positive control for maximal ROS generation. ROS generation was measured for 1 h using Diogenes chemiluminescence assay. ROS outputs are expressed on the *Y* axis as summarized RLU for 1 h normalized on the PMA signal as 100%. Five independent experiments, mean ± standard error. * *p* = 0.0221. MOI, multiplicity of infection (virus/PMN ratio); ns, nonsignificant; PMA, phorbol myristate acetate; RLU, relative luminescence unit; ROS, reactive oxygen species; Un, unstimulated.

We next assessed the effect of SARS-CoV-2 S1 proteins on ROS production in the presence of IAV in PMNs. PMNs were stimulated by different combinations of SARS-CoV-2 S1 proteins, anti-S IgG1, ICs, and IAV, and ROS production was measured. We used 10 μg/mL (the highest previously tested dose) of each S1 protein, 10 μg/mL of anti-S IgG1, or 10 μg/mL of each component for ICs formation and 1 MOI of influenza virus to PMN stimulation. No significant differences could be observed between PMNs stimulated by any of the S1 proteins alone, anti-S IgG1, ICs, or influenza versus unstimulated PMNs (**Figure 4**). Only IAV alone increased ROS production in PMNs, as expected (**Figure 4**). Thus, influenza-stimulated ROS production was not exaggerated but diminished by co-stimulation with S1 proteins, an anti-S1 IgG1 or their ICs.

**Figure 5:**
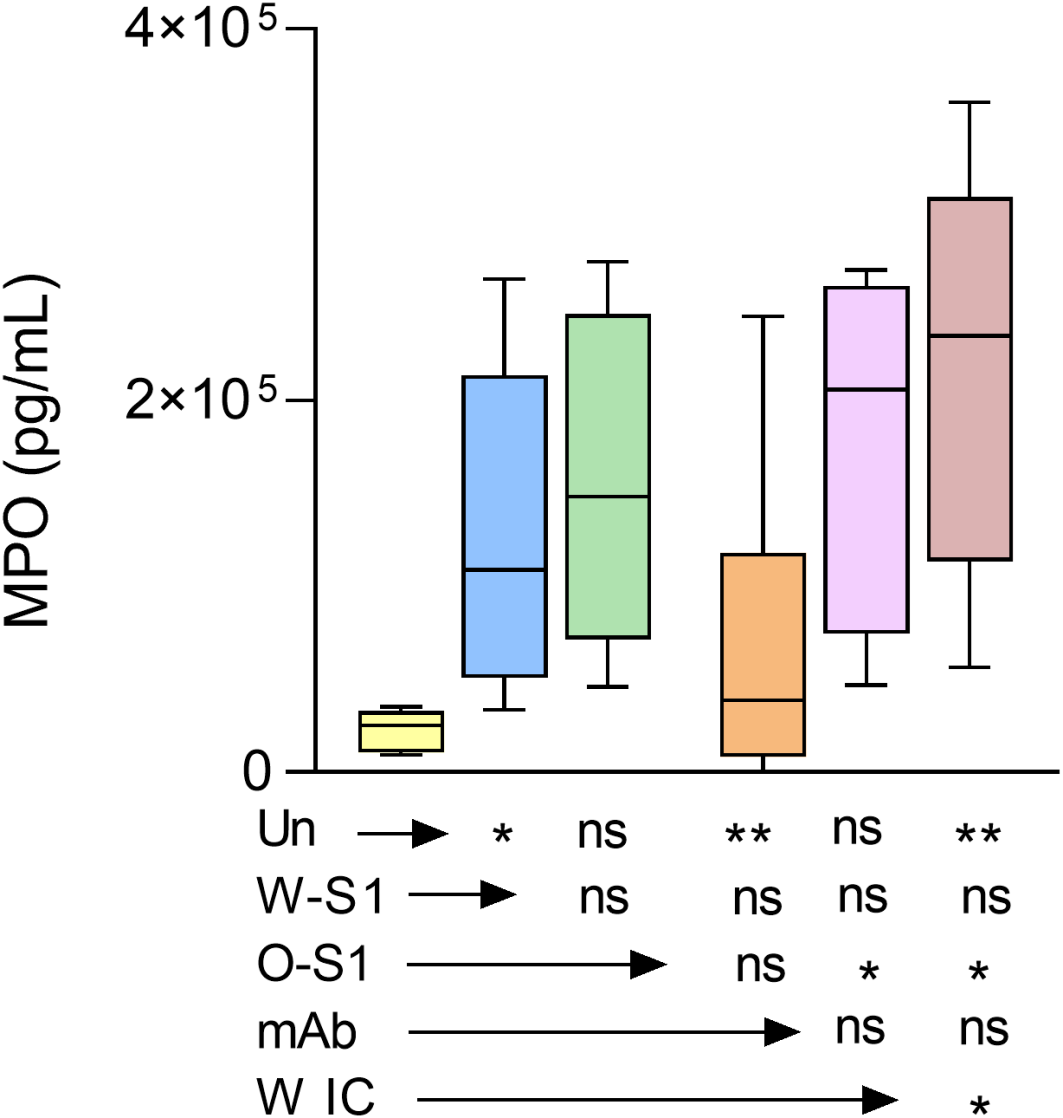
MPO release from PMNs stimulated with SARS-CoV-2 Wuhan and Omicron S1 proteins, anti-S IgG1, and ICs. Human PMNs were stimulated with the indicated stimuli and MPO release was measured by ELISA. The Wuhan spike protein (W-S1, 10 μg/mL of protein), the anti-S IgG1 (10 μg/mL, mAb), and the Omicron IC (O-IC, 10 μg/mL of each component) enhanced MPO production by PMNs (*n* = 6). However, no significant differences could be observed between the stimulation with Omicron S1 (O-S1) or Wuhan immune complex (W-IC) versus unstimulated PMNs. *, p<0.05; **, p<0.01. Un, unstimulated; ns, nonsignificant.

MPO is highly abundant in PMNs and generates the most reactive ROS, hypochlorous acid (HOCl) (16). MPO has also been associated with worse clinical outcomes in COVID-19 patients (17). Therefore, our next goal was to measure MPO release from PMNs stimulated with S1 proteins, anti-S antibody or their ICs. MPO levels in the PMN supernatants were quantified by ELISA. We observed that the Wuhan S1 protein, anti-S IgG1, and Omicron IC significantly enhanced MPO production by PMNs compared to the negative control. However, no significant differences were observed between stimulated versus unstimulated PMNs (**Figure 5**).

**Figure 6:**
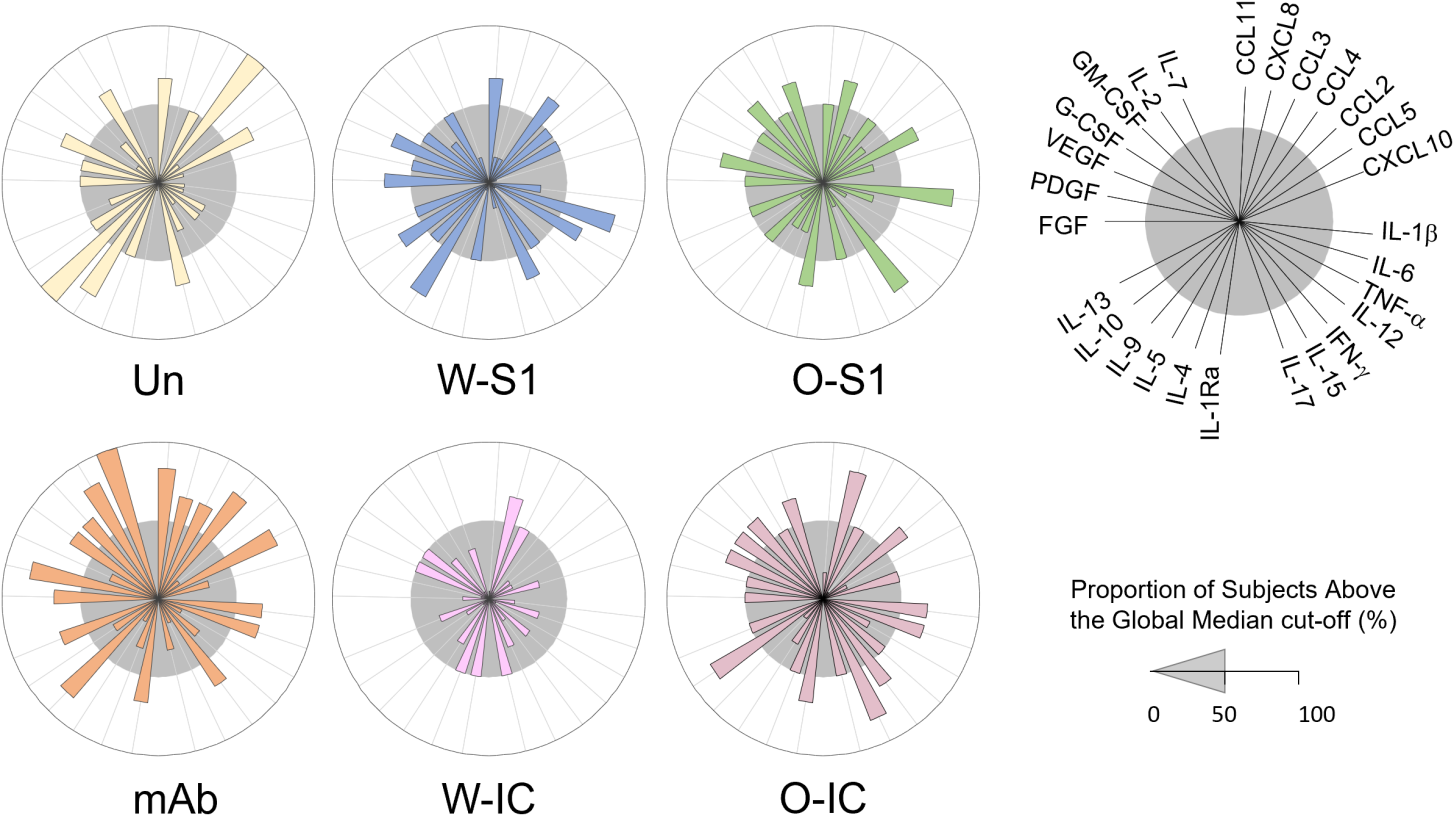
Levels of chemokines, cytokines, and growth factors in supernatants of human PMNs stimulated with S1 proteins or anti-S1 IgG1. Human PMNs were stimulated with Wuhan (W-S1) or Omicron (O-S1) spike proteins, anti-S1 IgG1, or their immune complexes (W-IC and O-IC) (each at 10 μg/mL concentrations), and the supernatants were collected and assayed for the levels of several cytokines and growth factors as indicated using a Luminex multiplex chemokine/cytokine/growth factors kit. Overall signature of soluble mediators in PMN supernatants was assembled by converting the raw data, expressed in pg/mL into categorical results to determine the proportion of subjects above the global median cutoff calculated for each analyte. Increased proportion of subjects was considered for values above the 50th percentile. Differences were observed between the different conditions and unstimulated PMNs. Un, unstimulated.

To better understand the responses of PMNs to SARS-CoV-2 infection, we also investigated the soluble mediator profile (chemokines, cytokines, and growth factors) released by PMNs. We quantified 27 analytes in culture supernatants of PMNs stimulated with each indicated stimuli and compared the responses to unstimulated PMNs. The results are presented as median concentration pg/mL (**Supplementary Table 1**) and also as overall signatures assembled as the proportion of subjects above the global median cutoff calculated for each analyte (**Figure 6**). We observed that the stimulation in culture supernatants induced different production of chemokines, proinflammatory and regulatory cytokines, as well as growth factors.

Overall, stimulus-dependent signatures were observed in the released cytokine profile of PMNs. The analysis of soluble mediators (expressed in pg/mL) demonstrated that the most differences were observed upon stimulation with W-IC and O-IC, with W-IC leading to a more pronounced decrease in several soluble mediators (**Supplementary Table 1**). Additional analysis, carried out after converting the data expressed in pg/mL into categorical data (proportion of subjects above the global median cutoff), is presented in **Figure 6**. We observed that the W-S1 increased levels of CCL11, CCL4, IL-6, TNF-α, IL-15, IL-5, IL-10, FGF-basic, and VEGF as observed by the proportion of subjects above the 50th percentile (**Figure 6**, gray circle). Our data also show that the O-S1 increased the levels of CXCL8, CCL5, IL-1*β*, IFN-γ, IL-1Ra, PDGF, GM-CSF, and IL-7. The stimulation with the anti-S1 IgG1 induced the production of CCL11, CXCL8, CCL3, CCL4, CCL5, IL-1*β*, IL-6, IFN-γ, IL-1Ra, IL-9, IL-13, FGF-basic, PDGF, G-CSF, GM-CSF, IL-2, and IL-7 (**Figure 6**). W-IC induces and increases only in CXCL8 production. Higher levels of CXCL8, CCL2, IL-1*β*, IL-6, IFN-γ, IL-1Ra, IL-15, IL-10, VEGF, G-CSF, GM-CSF, and IL-7 were found after the stimulation with O-IC (**Figure 6**).

## Discussion

Exploring the immune response is crucial for a better understanding of host–pathogen interactions. In the context of COVID-19, more studies are needed to investigate and comprehend the PMN response to SARS-CoV-2. Increasing amounts of data have been accumulating, showing that severe COVID-19 is marked by PMN abundance in the blood (18), altered physical PMN phenotypes —such as increased surface expression of CD11b and CD66b —demonstrating recent activation (19), and ARDS (20). PMNs play an essential role in ARDS and hyperactivated PMNs correlate with severe and fatal COVID-19 (20). MPO is an important element of PMNs (21). The MPO concentration is significantly elevated in COVID-19 patients who were admitted to a hospital intensive care unit (ICU) (22) and is associated with mortality and poor prognosis in COVID-19. MPO has been indicated as a potential prognostic marker for the severity of COVID-19 (17). MPO has also been associated with lung epithelial and endothelial damage in cases of severe COVID-19 (23), potentially accelerating oxidative lung tissue injuries in patients infected with SARS-CoV-2.

PMN activation is characterized by neutrophil extracellular trap (NET) formation, oxidative stress, release of antimicrobial peptides, and secretion of cytokines (24). An excessive generation of NETs resulting in a procoagulant profile (25), elevated levels of ROS and inflammatory mediators (19), and PMN degranulation (19) are described in SARS-CoV-2 infection and linked to COVID-19 disease severity. To better understand SARS-CoV-2 infection of PMNs, human PMNs from healthy donors were infected with the SARS-CoV-2 virus (26), its pseudovirus (27) and viral proteins (N, S, S1, S2) (28), and ICs derived from convalescent patients (29). Despite these few studies, however, it remains unclear how SARS- CoV-2 triggers PMN activation and which of the viral components are mainly responsible for this effect (30).

PMNs can be activated by pathogens’ molecules (24). Our data show no effect on ROS generation in PMNs stimulated with Wuhan S1 protein, similar to another study (28). We also analyzed the PMN response to the S1 protein of Omicron variant. Interestingly, our data demonstrate that Omicron S1 protein enhances ROS production by PMNs. The Omicron variant presents different immunogenicity and antigenicity compared to wild-type (Wuhan) SARS-CoV-2, and it is associated with mutations in the RBD region (27). Previous reports found that anti-SARS-CoV-2 antibodies may contribute to the exacerbation of the immune responses (31). Antibodies have the capacity of activating immune cells (32). High levels of IgG are frequently found in patients with severe COVID-19 and IgG1 is the most abundant subclass antibody in convalescent patients (33). The correlation between antibodies and PMN activation in COVID-19 is unknown. Previous data showed that anti-S IgG has been associated with acute lung injury by macrophage responses during acute SARS-CoV-2 infection (34). Our data show that anti-S IgG1 significantly induces ROS generation in human PMNs. Since PMNs have been linked to the pathophysiology of COVID-19, antibodies can be crucial activators of PMNs in COVID-19 *in vivo*, inducing an inflammatory response and oxidative stress. PMNs use FcγR receptors to bind the Fc region of IgG antibodies (35). The interaction between the SARS-CoV-2 spike protein RBD domain and the ACE2 receptor promotes virus entry into the host cell (36). Human PMNs express six classical FcγRs —FcγRI/CD64, FcγRIIA/CD32A, FcγRIIB/CD32B, FcγRIIC/CD32C, FcγRIIIA/CD16A, and FcγRIIIB/CD16B—and respond to monomeric or aggregated ICs, antigen-antibody ICs, and opsonized particles or cells (37, 38). In COVID-19, FcγR is reported to mediate SARS-CoV-2 infection of monocytes and lung macrophages and to enhance inflammation (39).

Different studies suggest that ICs have been associated with activation of cells such as PMNs, monocytes, and platelets (33). ICs have been correlated with a severe form of COVID- 19 (40) and shown to stimulate NET release and induce NETosis by PMNs (41). Since severe COVID-19 has been associated with elevated levels of antibodies and poor clinical outcomes, it raises the possibility of the contribution of ICs (41).

Our results suggest that ICs are involved in PMN activation in COVID-19. Our data also suggest that ICs might be involved in oxidative stress in COVID-19 since we detected significantly more ROS generation by PMNs when stimulated with the Wuhan IC. PMN activation and systemic inflammatory and thrombotic responses are associated with severe COVID-19 and ICs (35). A study showed that ICs were significantly higher in patients with severe COVID-19 compared with healthy donors (35), reinforcing the idea that ICs have an impact on COVID-19 pathogenesis. However, our findings indicate that Omicron IC does not significantly induce ROS generation by PMNs.

Other studies show that IgG (42) and different MOIs of SARS-CoV-2 (26) do not affect MPO release by PMNs. However, these studies did not use viral antigens and antiviral antibodies at the same time. We report here the association between ICs and MPO release. We observed that Wuhan ICs promote degranulation (specifically the release of MPO) in healthy human PMNs *in vitro*. Our finding shows that IAV significantly enhances ROS generation in PMNs, and it has been reported that IAV led to ROS production during infection, induced tissue injury, and cell death (43). The clinical impact of IAV and SARS-CoV-2 co-infection remains largely unknown. Studies showed that co-infection had less disease severity considering mortality (44). Our data show that PMNs stimulated with Wuhan and Omicron S1 proteins, anti-S antibody, or their ICs —in the presence or absence of IAV —do not enhance ROS production, suggesting that there is no hyperactivation of PMNs and excessive oxidative stress. In fact, the result suggests a rather inhibitory action of IAV infection on the oxidative response of PMNs to S1 protein, anti-S IgG1, or their IC, which could be a potential mechanism behind this latter observation.

We also observed that S1 proteins plus IAV and Omicron IC plus IAV significantly enhance MPO release from PMNs. Having anti-influenza IgM antibodies has been associated with significantly higher survival in COVID-19 patients (44). Our results indicate that the Wuhan S1 protein-IgG1 IC stimulated significantly higher MPO release from PMNs than unstimulated cells, while the other experimental conditions tested resulted in non-significant increases in MPO extrusion. Thus, the S1 protein is likely a significant contributor to MPO release from PMNs exposed to SARS-CoV-2, and different viral variants lead to different amounts of MPO released.

Interestingly, the presence of H3N2 influenza virus alone led to enhanced, although non- significant, MPO release and further promoted MPO release induced by the S1 proteins of both variants and the Omicron IC. These data indicate that MPO levels in COVID-19 patients might be exaggerated by influenza co-infection that can also further worsen PMN-mediated inflammation in the patients. Published studies found indeed that COVID-19 patients co-infected with other respiratory pathogens, mainly influenza viruses and enteroviruses, were more likely to be dyspneic and the odds of fatality (OR = 1.66) were also increased —even if a relatively low proportion of COVID-19 patients had a respiratory viral co-infection (45).

Chemokines, growth factors, and regulatory and proinflammatory cytokines are involved in the regulation of PMN function (46). Dysregulation of the inflammatory response has been associated with SARS-CoV-2 infection and severe cases of COVID-19 (46). PMN recruitment in COVID-19 is associated with cytokine storm (46). When COVID-19 progresses to severe illness, higher serum levels of IL-6, CXCL8, TNF-α, and IL-1β are associated with hyperinflammatory and hyper-coagulable conditions (47). Among cytokines, IL-6 and IL-1β were also associated with fever (47). Our data show that the levels of CXCL8 increased after incubation with O-S1, IgG1, W-IC, or O-IC. However, in supernatants stimulated with W-S1, IgG1 or O-IC induced higher levels of IL-6. Interestingly, our results also showed that only W-S1 increased TNF-α levels in PMN supernatants. In addition, we observed that O-S1, IgG1, or O-IC induced the production of IL-1β. According to the literature, higher levels of CCL2 and CCL3 also play a role in COVID-19 severity (48). Our research showed that O-IC increased the levels of CCL2. IFN-γ levels are elevated in patients with severe COVID-19 (49). Our results indicated that the levels of these inflammatory cytokines were increased by IgG1, O-S1, and O-IC. While we observed enhanced levels of several cytokines and growth factors following PMN stimulation, their signature is different among conditions and also associate with the type of the S1 variant.

In summary, our results show that human PMNs respond to the presence of the SARS- CoV-2 S1 protein with ROS production that is further complicated by the presence of anti-S1 IgG1 or influenza virus. Oxidative responses of PMNs also demonstrate variability depending on the SARS-CoV-2 variant. The data presented here help better understand the complex and clinically critical relationship between SARS-CoV-2 and PMNs.

## Data availability statement

The raw data supporting the conclusions of this article will be made available by the authors, without undue reservation.

## Ethics statement

The studies involving humans were approved by University of Georgia Institutional Review Board. The studies were conducted in accordance with the local legislation and institutional requirements. The participants provided their written informed consent to participate in this study.

## Author contributions

NBFA: Conceptualization, Data curation, Formal Analysis, Methodology, Validation, Visualization, Writing – original draft, Writing – review & editing. KF: Data curation, Writing – review & editing. DS: Data curation, Methodology, Writing – review & editing. NMA: Data curation, Writing – review & editing. SC: Data curation, Writing – review & editing. RG: Formal Analysis, Funding acquisition, Supervision, Visualization, Writing – review & editing. OAM-F: Formal Analysis, Visualization, Writing – review & editing. BR: Conceptualization, Formal Analysis, Funding acquisition, Resources, Supervision, Validation, Writing – review & editing.

## References

[1] Li Q Guan X Wu P Wang X Zhou L Tong Y. Early transmission dynamics in wuhan, China, of novel coronavirus-infected pneumonia. N Engl J Med (2020) 382(13):1199–207. doi: 10.1056/NEJMoa2001316 PMC7121484 31995857

[2] Gorbalenya AE Baker SC Baric RS de Groot RJ Drosten C Gulyaeva AA. V. Coronaviridae Study Group of the International Committee on Taxonomy of. The species Severe acute respiratory syndrome-related coronavirus: classifying 2019-nCoV and naming it SARS-CoV-2. Nat Microbiol (2020) 5(4):536–44. doi: 10.1038/s41564-020-0695-z PMC7095448 32123347

[3] Silva AAMD. On the possibility of interrupting the coronavirus (COVID-19) epidemic based on the best available scientific evidence. Rev Bras Epidemiol. (2020) 23:e200021. doi: 10.1590/1980-549720200021 32187257

[4] Chan JF Kok KH Zhu Z Chu H To KK Yuan S. Genomic characterization of the 2019 novel human-pathogenic coronavirus isolated from a patient with atypical pneumonia after visiting Wuhan. Emerg Microbes Infect (2020) 9(1):221–36. doi: 10.1080/22221751.2020.1719902 PMC7067204 31987001

[5] Shang J Ye G Shi K Wan Y Luo C Aihara H. Structural basis of receptor recognition by SARS-CoV-2. Nature (2020) 581(7807):221–4. doi: 10.1038/s41586-020-2179-y PMC7328981 32225175

[6] Oude Munnink BB Koopmans M. Tracking SARS-CoV-2 variants and resources. Nat Methods (2023) 20(4):489–90. doi: 10.1038/s41592-023-01833-y 36922622

[7] Ye G Liu B Li F. Cryo-EM structure of a SARS-CoV-2 omicron spike protein ectodomain. Nat Commun (2022) 13(1):1214. doi: 10.1038/s41467-022-28882-9 35241675 PMC8894419

[8] Yang SC Tsai YF Pan YL Hwang TL. Understanding the role of neutrophils in acute respiratory distress syndrome. BioMed J (2021) 44(4):439–46. doi: 10.1016/j.bj.2020.09.001 PMC7481802 33087299

[9] Laforge M Elbim C Frère C Hémadi M Massaad C Nuss P. Tissue damage from neutrophil-induced oxidative stress in COVID-19. Nat Rev Immunol (2020) 20(9):515–6. doi: 10.1038/s41577-020-0407-1 PMC7388427 32728221

[10] Faurschou M Borregaard N. Neutrophil granules and secretory vesicles in inflammation. Microbes Infect (2003) 5(14):1317–27. doi: 10.1016/j.micinf.2003.09.008 14613775

[11] Pongracz T Nouta J Wang W van Meijgaarden KE Linty F Vidarsson G. Immunoglobulin G1 Fc glycosylation as an early hallmark of severe COVID-19. EBioMedicine (2022) 78:103957. doi: 10.1016/j.ebiom.2022.103957 35334306 PMC8938159

[12] Gasparoto TH Dalboni TM Amôr NG Abe AE Perri G Lara VS. Fcγ receptors on aging neutrophils. J Appl Oral Sci (2021) 29:e20200770. doi: 10.1590/1678-7757-2020-0770 33825754 PMC8011831

[13] Hoepel W Chen HJ Geyer CE Allahverdiyeva S Manz XD de Taeye SW. High titers and low fucosylation of early human anti-SARS-CoV-2 IgG promote inflammation by alveolar macrophages. Sci Transl Med (2021) 13(596):eabf8654. doi: 10.1126/scitranslmed.abf8654 33979301 PMC8158960

[14] Lampejo T. Influenza and antiviral resistance: an overview. Eur J Clin Microbiol Infect Dis (2020) 39(7):1201–8. doi: 10.1007/s10096-020-03840-9 PMC7223162 32056049

[15] Roberts NJ Jr Krilov LR. The continued threat of influenza A viruses. Viruses (2022) 14(5):883. doi: 10.3390/v14050883 35632626 PMC9143665

[16] Winterbourn CC Kettle AJ Hampton MB. Reactive oxygen species and neutrophil function. Annu Rev Biochem (2016) 85:765–92. doi: 10.1146/annurev-biochem-060815-014442 27050287

[17] Ouwendijk WJD Raadsen MP van Kampen JJA Verdijk RM von der Thusen JH Guo L. High levels of neutrophil extracellular traps persist in the lower respiratory tract of critically ill patients with coronavirus disease 2019. J Infect Dis (2021) 223(9):1512–21. doi: 10.1093/infdis/jiab050 PMC7928833 33507309

[18] Eastin C Eastin T. Clinical Characteristics of Coronavirus Disease 2019 in China: Guan W, Ni Z, Hu Y, et al. N Engl J Med. 2020 Feb 28 [Online ahead of print] DOI: 10.1056/NEJMoa2002032. J Emerg Med (2020) 58(4):711–2. doi: 10.1016/j.jemermed.2020.04.004

[19] Reusch N De Domenico E Bonaguro L Schulte-Schrepping J Baßler K Schultze JL. Neutrophils in COVID-19. Front Immunol (2021) 12:652470. doi: 10.3389/fimmu.2021.652470 33841435 PMC8027077

[20] Wang D Hu B Hu C Zhu F Liu X Zhang J. Clinical characteristics of 138 hospitalized patients with 2019 novel coronavirus-infected pneumonia in wuhan, China. JAMA (2020) 323(11):1061–9. doi: 10.1001/jama.2020.1585 PMC7042881 32031570

[21] Davies MJ Hawkins CL. The role of myeloperoxidase in biomolecule modification, chronic inflammation, and disease. Antioxid Redox Signal (2020) 32(13):957–81. doi: 10.1089/ars.2020.8030 31989833

[22] Peyneau M Granger V Wicky PH Khelifi-Touhami D Timsit JF Lescure FX. Innate immune deficiencies are associated with severity and poor prognosis in patients with COVID-19. Sci Rep (2022) 12(1):638. doi: 10.1038/s41598-021-04705-7 35022495 PMC8755788

[23] Guéant JL Guéant-Rodriguez RM Fromonot J Oussalah A Louis H Chery C. Elastase and exacerbation of neutrophil innate immunity are involved in multi-visceral manifestations of COVID-19. Allergy (2021) 76(6):1846–58. doi: 10.1111/all.14746 PMC8014109 33484168

[24] Mayadas TN Cullere X Lowell CA. The multifaceted functions of neutrophils. Annu Rev Pathol (2014) 9:181–218. doi: 10.1146/annurev-pathol-020712-164023 24050624 PMC4277181

[25] Janiuk K Jabłońska E Garley M. Significance of NETs formation in COVID-19. Cells (2021) 10(1):151. doi: 10.3390/cells10010151 33466589 PMC7828704

[26] Muralidharan A Wyatt TA Reid SP. SARS-coV-2 dysregulates neutrophil degranulation and reduces lymphocyte counts. Biomedicines (2022) 10(2):382. doi: 10.3390/biomedicines10020382 35203591 PMC8962352

[27] Li Q Zhang M Liang Z Zhang L Wu X Yang C. Antigenicity comparison of SARS-CoV-2 Omicron sublineages with other variants contained multiple mutations in RBD. MedComm (2020). (2022) 3(2):e130. doi: 10.1002/mco2.130 35434713 PMC8994617

[28] Youn YJ Lee YB Kim SH Jin HK Bae JS Hong CW. Nucleocapsid and spike proteins of SARS-coV-2 drive neutrophil extracellular trap formation. Immune Netw (2021) 21(2):e16. doi: 10.4110/in.2021.21.e16 33996172 PMC8099611

[29] Jevtic SD Nazy I. The COVID complex: A review of platelet activation and immune complexes in COVID-19. Front Immunol (2022) 13:807934. doi: 10.3389/fimmu.2022.807934 35371058 PMC8965558

[30] Del Valle DM Kim-Schulze S Huang HH Beckmann ND Nirenberg S Wang B. An inflammatory cytokine signature predicts COVID-19 severity and survival. Nat Med (2020) 26(10):1636–43. doi: 10.1038/s41591-020-1051-9 PMC7869028 32839624

[31] Luo H Jia T Chen J Zeng S Qiu Z Wu S. The characterization of disease severity associated igG subclasses response in COVID-19 patients. Front Immunol (2021) 12:632814. doi: 10.3389/fimmu.2021.632814 33763078 PMC7982848

[32] Rosales C Uribe-Querol E. Neutrophil activation by antibody receptors. Neutrophils [Internet]. (2018) Nov 5 [cited 2022 Jun 7].

[33] Jarlhelt I Nielsen SK Jahn CXH Hansen CB Pérez-Alós L Rosbjerg A. SARS-coV-2 antibodies mediate complement and cellular driven inflammation. Front Immunol (2021) 12:767981. doi: 10.3389/fimmu.2021.767981 34804055 PMC8596567

[34] Liu L Wei Q Lin Q Fang J Wang H Kwok H. Anti-spike IgG causes severe acute lung injury by skewing macrophage responses during acute SARS-CoV infection. JCI Insight (2019) 4(4):e123158. doi: 10.1172/jci.insight.123158 30830861 PMC6478436

[35] Mazzitelli I Bleichmar L Ludueña MG Pisarevsky A Labato M Chiaradia V. Immunoglobulin G immune complexes may contribute to neutrophil activation in the course of severe coronavirus disease 2019. J Infect Dis (2021) 224(4):575–85. doi: 10.1093/infdis/jiab174 PMC8083460 34398243

[36] Hoffmann M Kleine-Weber H Schroeder S Krüger N Herrler T Erichsen S. SARS-coV-2 cell entry depends on ACE2 and TMPRSS2 and is blocked by a clinically proven protease inhibitor. Cell (2020) 181(2):271–280.e8. doi: 10.1016/j.cell.2020.02.052 32142651 PMC7102627

[37] Bruhns P Iannascoli B England P Mancardi DA Fernandez N Jorieux S. Specificity and affinity of human Fcgamma receptors and their polymorphic variants for human IgG subclasses. Blood (2009) 113(16):3716–25. doi: 10.1182/blood-2008-09-179754 19018092

[38] Wang Y Jönsson F. Expression, role, and regulation of neutrophil fcγ Receptors. Front Immunol (2019) 10:1958. doi: 10.3389/fimmu.2019.01958 31507592 PMC6718464

[39] Junqueira C Crespo Â Ranjbar S de Lacerda LB Lewandrowski M Ingber J. FcγR-mediated SARS-CoV-2 infection of monocytes activates inflammation. Nature (2022) 606(7914):576–84. doi: 10.1038/s41586-022-04702-4 PMC10071495 35385861

[40] Park YJ Acosta D Vassell R Tang J Khurana S Weiss CD. D-dimer and CoV-2 spike-immune complexes contribute to the production of PGE2 and proinflammatory cytokines in monocytes. PloS Pathog (2022) 18(4):e1010468. doi: 10.1371/journal.ppat.1010468 35385545 PMC9015149

[41] Behzadifard M Soleimani M. NETosis and SARS-COV-2 infection related thrombosis: a narrative review. Thromb J (2022) 20(1):13. doi: 10.1186/s12959-022-00375-1 35354492 PMC8965217

[42] Bartsch YC Wang C Zohar T Fischinger S Atyeo C Burke JS. Humoral signatures of protective and pathological SARS-CoV-2 infection in children. Nat Med (2021) 27(3):454–62. doi: 10.1038/s41591-021-01263-3 PMC8315827 33589825

[43] Chen KK Minakuchi M Wuputra K Ku CC Pan JB Kuo KK. Redox control in the pathophysiology of influenza virus infection. BMC Microbiol (2020) 20(1):214. doi: 10.1186/s12866-020-01890-9 32689931 PMC7370268

[44] Ding Q Lu P Fan Y Xia Y Liu M. The clinical characteristics of pneumonia patients coinfected with 2019 novel coronavirus and influenza virus in Wuhan, China. J Med Virol (2020) 92(9):1549–55. doi: 10.1002/jmv.25781 PMC7228290 32196707

[45] Dao TL Hoang VT Colson P Million M Gautret P. Co-infection of SARS-CoV-2 and influenza viruses: A systematic review and meta-analysis. J Clin Virol Plus. (2021) 1(3):100036. doi: 10.1016/j.jcvp.2021.100036 35262019 PMC8349735

[46] Sahanic S Löffler-Ragg J Tymoszuk P Hilbe R Demetz E Masanetz RK. The role of innate immunity and bioactive lipid mediators in COVID-19 and influenza. Front Physiol (2021) 12:688946. doi: 10.3389/fphys.2021.688946 34366882 PMC8339726

[47] Guo J Wang S Xia H Shi D Chen Y Zheng S. Cytokine signature associated with disease severity in COVID-19. Front Immunol (2021) 12:681516. doi: 10.3389/fimmu.2021.681516 34489933 PMC8418386

[48] Hirano T Murakami M. COVID-19: A new virus, but a familiar receptor and cytokine release syndrome. Immunity (2020) 52(5):731–3. doi: 10.1016/j.immuni.2020.04.003 PMC7175868 32325025

[49] Gadotti AC de Castro Deus M Telles JP Wind R Goes M Garcia Charello Ossoski R. IFN-γ is an independent risk factor associated with mortality in patients with moderate and severe COVID-19 infection. Virus Res (2020) 289:198171. doi: 10.1016/j.virusres.2020.198171 32979474 PMC7510544

