# Supplementary material for: Variant-dependent oxidative and cytokine responses of human neutrophils to SARS-CoV-2 spike protein and anti-spike IgG1 antibodies
Source: Front Immunol. 2023 Oct 16;14:1255003. doi: 10.3389/fimmu.2023.1255003 (PMC10613679; doi:10.3389/fimmu.2023.1255003)
Supplement: Supplementary file 1 [file DataSheet_1.pdf]

Supplementary Table 1. Concentration of chemokines, proinflammatory cytokines, regulatory cytokines and growth factors in PMN supernatants upon *in vitro* culture.

Analytes	*In vitro* Stimuli					
	Un	W-S1	O-S1	IgG1	W-IC	O-IC
CCL11	26 (25-26)	27 (24-29)	26 (24-28)	29 (27-33)	21 (20-22) [a,c,d]	20[e] (19-23) [a,c,d]
CXCL8	96 (70-163)	212 (184-250)	476 (252-765) [a]	331 (235-427) [a]	448 (292-602) [a,b]	600 (419-664) [a,b]
CCL3	15 (12-16)	13 (13-13)	14 (12-16)	16 (14-18)	15 (14-15)	15 (13-17)
CCL4	116 (93-136)	93 (59-122)	82 (52-116)	128 (109-131)	45 (37-50) [a,c,d]	46 (44-55) [a,c,d]
CCL2	16 (15-17)	18 (17-18)	17 (17-18)	16 (15-17)	17 (16-17)	19 (17-20)
CCL5	327 (280-367)	306 (274-321)	309 (306-346)	427 (344-529)	81 (53-108) [a,d]	45 (38-125) [a,c,d]
CCL10	12 (10-12)	11 (10-12)	11 (10-12)	12 (12-13)	12 (10-12)	14 (12-15) [b]
IL-1β	16 (14-16)	15 (14-18)	19 (17-21) [a]	18 (16-19)	16 (14-16)	20 (17-22)
IL-6	20 (18-20)	23 (21-26)	19 (17-21)	21 (19-23)	20 (20-22)	21 (19-23)
TNF-α	20 (19-20)	24 (21-27) [a]	19 (16-20) [b]	20 (19-20) [b]	20 (19-20) [b]	20 (19-22) [b]
IL-12	19 (18-19)	18 (17-18)	18 (17-18)	19 (19-20) [b]	19 (18-20)	19 (19-20) [b]
IFN-γ	26 (25-28)	30 (26-33)	37 (34-38) [a,b]	37 (29-40) [a]	29 (27-31) [c]	32 (31-35) [a]
IL-15	23 (22-27)	29 (26-33)	24 (21-25)	23 (22-23)	25 (21-27)	28 (26-30)
IL-17	19 (18-20)	18 (17-18)	18 (17-20)	18 (17-19)	18 (17-19)	17 (17-21)
IL-1Ra	35 (33-46)	188 (81-271)	458 (249-1070) [a]	300 (139-377) [a]	245 (126-320) [a]	500 (141-506) [a]
IL-4	9 (9-10)	9 (9-9)	9 (8-10)	9 (8-9)	9 (8-10)	10 (8-12)
IL-5	37 (37-37)	37 (35-39)	27 (22-33) [a,b]	33 (31-34) [a]	31 (27-35)	27 (25-35)
IL-9	46 (45-46)	36 (30-42)	34 (27-51)	46 (38-52)	25 (23-27) [a,d]	28 (23-29) [a,c,d]
IL-10	18 (17-19)	18 (16-18)	17 (15-17)	17 (16-18)	16 (16-17)	20 (18-21) [e]
IL-13	11 (10-12)	11 (10-12)	11 (8-13)	12 (11-13)	11 (10-12)	11 (10-15)
FGF-basic	15 (13-16)	16 (14-19)	16 (13-19)	17 (15-19)	14 (13-14)	16 (13-18)
PDGF	31 (29-32)	31 (28-36)	31 (30-33)	38 (33-48)	24 (23-26) [a,b,c,d]	31 (25-33) [c,d]
VEGF	59 (53-64)	63 (53-76) [b,c]	48 (43-50)	49 (44-63)	53 (50-55)	56 (52-57)
G-CSF	25 (24-26)	29 (25-31)	27 (25-30)	31 (28-36) [a]	27 (25-27) [d]	29 (26-31) [c,d]
GM-CSF	25 (24-28)	24 (23-26)	27 (25-28)	27 (23-28)	24 (23-27)	28 (26-28)
IL-2	21 (20-22)	21 (19-21)	21 (18-22)	21 (21-22)	19 (18-19) [a,d]	19 (18-22) [c,d]
IL-7	62 (59-63)	63 (60-65)	67 (63-69)	69 (67-74) [a,b]	64 (62-67)	68 (65-68) [a]

Human PMNs were stimulated with Wuhan (W-S1) or Omicron (O-S1) spike proteins, anti-S1 IgG1, or their immune complexes (W-IC, O-IC) (each at 10µg/mL concentrations), the supernatants were collected and assayed for the levels of chemokines, proinflammatory cytokines, regulatory cytokines and growth factors using a Luminex multiplex kit as described in Material and Methods. The results are presented as median values and interquartile range (25th-75th IQR) of analyte concentration (pg/mL) measured in PMNs supernatants. The letters "a", "b", "c", "d" and "e" indicate significant differences as compared to unstimulated, W-S1, O-S1, IgG1, W-IC and O-IC, respectively.
