# Supplementary material for: Variant-dependent oxidative and cytokine responses of human neutrophils to SARS-CoV-2 spike protein and anti-spike IgG1 antibodies
Source: Front Immunol. 2023 Oct 16;14:1255003. doi: 10.3389/fimmu.2023.1255003 (PMC10613679; doi:10.3389/fimmu.2023.1255003)
Supplement: Supplementary file 2 [file DataSheet_2.pdf]

Supplementary figure 1.

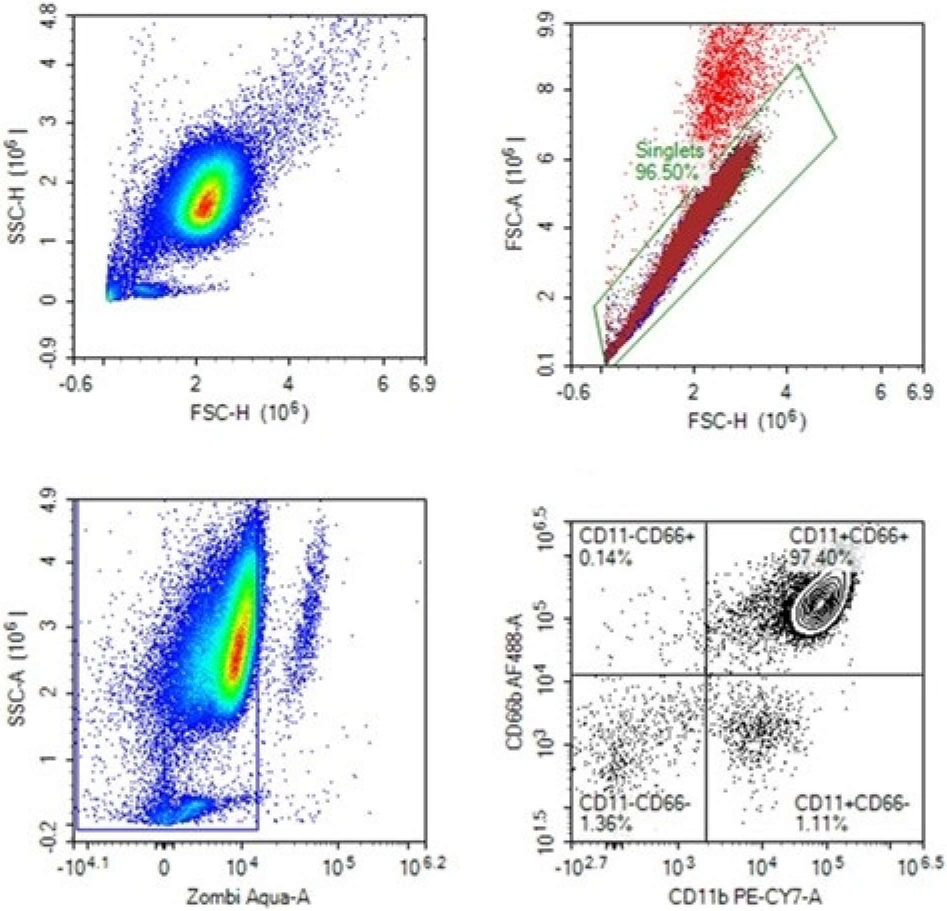

Representative flow cytometry plots of isolated $CD11b^{+}/CD66b^{+}$ live PMNs from human peripheral blood. The overall profile of PMNs was first monitored on forward scatter height (FSC-H) vs side scatter height (SSC-H) pseudocolor density plots followed by the selection singlets using forward scatter area (FSC-A) *vs* forward scatter height (FSC-H) dot plot distribution. PMNs viability was assessed using Zombie Aqua-A fluorescence *vs* side scatter area (SSC-A) quadrant statistics on pseudocolor density plots. PMNs purity was determined by quadrant statistics on $CD11b^{+}$ vs $CD66b^{+}$ contours plots. Overall, the protocol resulted in PMNs preparation with ~95% purity.
